# Exploring Radioiodinated Anastrozole and Epirubicin as AKT1-Targeted Radiopharmaceuticals in Breast Cancer: In Silico Analysis and Potential Therapeutic Effect with Functional Nuclear Imagining Implications

**DOI:** 10.3390/molecules29174203

**Published:** 2024-09-04

**Authors:** Mazen Abdulrahman Binmujlli

**Affiliations:** Department of Internal Medicine, College of Medicine, Imam Mohammad Ibn Saud Islamic University (IMSIU), P.O. Box 90950, Riyadh 11623, Saudi Arabia; dr1mazen@gmail.com

**Keywords:** breast cancer, AKT1, molecular docking, molecular dynamic, MM-PBSA

## Abstract

This study evaluates radio-iodinated anastrozole ([^125^I]anastrozole) and epirubicin ([^125^I]epirubicin) for AKT1-targeted breast cancer therapy, utilizing radiopharmaceutical therapy (RPT) for personalized treatment. Through molecular docking and dynamics simulations (200 ns), it investigates these compounds’ binding affinities and mechanisms to the AKT1 enzyme, compared to the co-crystallized ligand, a known AKT1 inhibitor. Molecular docking results show that [^125^I]epirubicin has the highest Δ*G*_bind_ (−11.84 kcal/mol), indicating a superior binding affinity compared to [^125^I] anastrozole (−10.68 kcal/mol) and the co-crystallized ligand (−9.53 kcal/mol). Molecular dynamics (MD) simulations confirmed a stable interaction with the AKT1 enzyme, with [^125^I]anastrozole and [^125^I]epirubicin reaching stability after approximately 68 ns with an average RMSD of around 2.2 Å, while the co-crystallized ligand stabilized at approximately 2.69 Å after 87 ns. RMSF analysis showed no significant shifts in residues or segments, with consistent patterns and differences of less than 2 Å, maintaining enzyme stability. The [^125^I]epirubicin complex maintained an average of four H-bonds, indicating strong and stable interactions, while [^125^I]anastrozole consistently formed three H-bonds. The average Rg values for both complexes were ~16.8 ± 0.1 Å, indicating no significant changes in the enzyme’s compactness, thus preserving structural integrity. These analyses reveal stable binding and minimal structural perturbations, suggesting the high potential for AKT1 inhibition. MM-PBSA calculations confirm the potential of these radio-iodinated compounds as AKT1 inhibitors, with [^125^I]epirubicin exhibiting the most favorable binding energy (−23.57 ± 0.14 kcal/mol) compared to [^125^I]anastrozole (−20.03 ± 0.15 kcal/mol) and the co-crystallized ligand (−16.38 ± 0.14 kcal/mol), highlighting the significant role of electrostatic interactions in stabilizing the complex. The computational analysis shows [^125^I]anastrozole and [^125^I]epirubicin may play promising roles as AKT1 inhibitors, especially [^125^I]epirubicin for its high binding affinity and dynamic receptor interactions. These findings, supported by molecular docking scores and MM-PBSA binding energies, advocate for their potential superior inhibitory capability against the AKT1 enzyme. Nevertheless, it is crucial to validate these computational predictions through in vitro and in vivo studies to thoroughly evaluate the therapeutic potential and viability of these compounds for AKT1-targeted breast cancer treatment.

## 1. Introduction

Breast cancer is one of the most prevalent malignancies affecting women worldwide, accounting for a significant number of cancer-related deaths each year [1]. According to the World Health Organization (WHO), breast cancer is the most common cancer among women, with an estimated 2.3 million new cases diagnosed in 2020 alone [2,3]. The global burden of breast cancer continues to rise, partly due to increasing life expectancy, urbanization, and the adoption of Western lifestyles [4]. Despite advances in therapeutic strategies, early and accurate diagnoses remain a critical challenge [5,6]. The five-year survival rate for breast cancer patients is significantly higher when the disease is detected at an early stage, underscoring the importance of effective diagnostic tools [7].

Mammography has been the gold standard for breast cancer screening; however, its sensitivity and specificity can be limited, particularly in women with dense breast tissue [8]. In recent years, molecular imaging techniques have gained prominence in the field of oncology, offering enhanced sensitivity and specificity for detecting malignancies [9,10]. Radiopharmaceuticals, which are radioactive compounds used for diagnosis and therapy, have emerged as valuable tools for imaging and targeting tumors [11,12]. These compounds can provide detailed information about the biological processes underlying cancer, facilitating early detection and personalized treatment strategies [11,12].

The integration of radiopharmaceuticals into clinical practice holds significant potential to transform breast cancer management [13]. Radiopharmaceuticals are employed in advanced imaging techniques, such as positron emission tomography (PET) and single-photon emission computed tomography (SPECT), to capture and visualize the metabolic and molecular processes occurring within tumors [14,15,16]. These techniques provide functional imaging capabilities, enabling clinicians to assess tumor metabolism, proliferation, and receptor status, which are essential for developing personalized treatment strategies [14,15,16]. In breast cancer, hormonal receptors, like estrogen (ER), progesterone (PR), and androgens (ARs), serve as crucial targets for the non-invasive whole-body evaluation of hormonal status, helping to predict cancer response to endocrine treatments [13,17]. A notable example is 16α-[^18^F]-fluoro-17β-estradiol (FES), a radiopharmaceutical widely used for the PET imaging of ER expression [18]. Additionally, radiolabeled monoclonal antibody trastuzumab, applicable in both SPECT and PET, facilitates the imaging of the human epidermal growth factor receptor 2 (HER2) [19,20,21].

**Figure 1:**
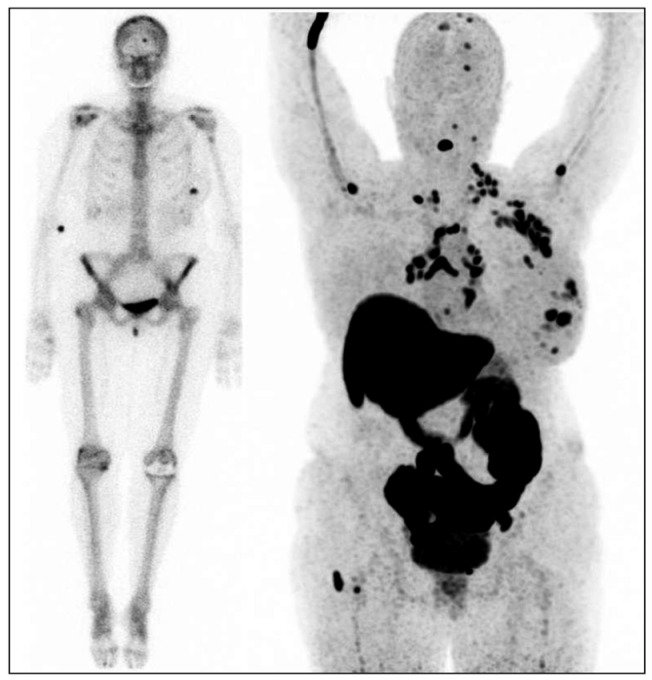
Female patient with the estrogen receptor (ER)-positive breast cancer in her left breast. Left image: bone scan for dissemination: uptake in the primary tumor in the left breast and uptake in the skull [13]. Right image: [^18^F]-FES-PET with multiple lesions in the left breast, multiple ER-positive lymph node metastases (axillae, clavicular regions, neck, mediastinum, and hili), and multiple ER-positive bone metastases (skull, spine, left humerus, and right femur).

Molecular imaging with [^18^F]-fluoroestradiol positron emission tomography ([^18^F]-FES-PET) offers non-invasive insights into ER status across all metastases within a patient’s body [22]. This technique is valuable for assessing the ER binding of endocrine modulators or down-regulators by comparing ER status before and during therapy [17]. Preclinical studies have demonstrated a strong correlation between [^18^F]-FES uptake and ER expression as determined by immunohistochemistry, with [^18^F]-FES uptake levels predicting the response to hormonal therapy (Figure 1) [23,24]. Recent studies have reported an overall sensitivity of 84% and specificity of 98% for [^18^F]-FES-PET in breast cancer patients, with a Standard Uptake Value max (SUVmax) threshold of 1.5 used to predict therapeutic response; values below this threshold generally indicate the lack of a response to endocrine therapy [25,26,27]. Moreover, [^18^F]-FES-PET proves useful for clinical dilemmas where conventional imaging and biopsies are inconclusive or impractical, aiding in therapy decisions by enhancing diagnostic clarity [28,29]. This imaging modality is also beneficial for monitoring ER status in progressive disease, thereby informing the continuation of or alteration in anti-hormonal treatments [18]. For therapy evaluation, [^18^F]-FES-PET can detect treatment response significantly earlier than traditional CT scans, potentially guiding the timely adjustments to therapy [30,31,32]. Although primarily visualizing ERα, [^18^F]-FES-PET has also shown efficacy in assessing ER status in gynecological tumors predominantly expressing ERβ, with ongoing efforts to develop specific ERβ tracers [33,34].

In addition to these advancements, radiopharmaceuticals have shown potential utility in managing triple-negative breast cancer (TNBC), an aggressive subtype characterized by the absence of ER, PR, and HER2 receptors, which limits the efficacy of conventional targeted therapies [35,36,37]. Radiopharmaceuticals targeting other molecular markers or metabolic pathways specific to TNBC could provide new therapeutic avenues, especially in cases where traditional treatments are ineffective [35,36,37]. This highlights the broader applicability of radiopharmaceuticals beyond receptor-positive breast cancers, underscoring their potential for addressing challenging cancer subtypes, like TNBC [35,36,37].

Targeting key signaling pathways in cancer cells has shown promise for improving diagnostic and therapeutic outcomes [38]. The PI3K/AKT/mTOR signaling pathway is a critical regulator of cell growth, proliferation, survival, and metabolism, and its dysregulation is commonly observed in breast cancer [39,40]. AKT1, a serine/threonine kinase, is a pivotal component of this pathway and represents a compelling target for anticancer therapy [41,42]. Aberrant activation of AKT1 has been associated with cancer progression, metastasis, and resistance to therapy, particularly in breast cancer [43,44]. Inhibiting AKT1 can suppress tumor growth, induce apoptosis, and enhance the efficacy of existing treatments by sensitizing cancer cells to chemotherapy and radiotherapy [45]. A notable example is ARQ092 (Miransertib), a selective allosteric inhibitor of AKT1, which has shown promise in clinical trials for patients with PI3K/Akt-driven tumors or Proteus syndrome [46]. Therefore, targeting AKT1 could have a significant impact on improving the treatment outcomes for breast cancer patients. Recent studies have highlighted the potential of radio-iodinated compounds, such as anastrozole and epirubicin, in the field of oncology [47,48]. Radioactive iodine attached to an sp3 carbon atom is a rare feature in radiopharmaceutical compounds; however, some have proven effective in treating solid tumors [49]. For instance, iododoxorubicin has demonstrated significant advancements, showcasing the potential of radio-iodinated derivatives in cancer treatment [50,51,52]. Studies on iododoxorubicin have demonstrated enhanced tumor uptake and an improved pharmacokinetic profile compared to its non-radio-iodinated variants [50,51,52]. In this context, radio-iodinated anastrozole ([^125^I]anastrozole) and radio-iodinated epirubicin ([^125^I]epirubicin) (Figure S1) have been studied as potential targeting radiopharmaceuticals for solid tumor imaging [47].

Although [^125^I]anastrozole and [^125^I]epirubicin demonstrate significant potential in targeting solid tumors, elucidating their interactions with enzymes remains a complex challenge. Traditional methodologies for evaluating enzyme inhibition are often both intricate and resource intensive [53,54]. In this regard, computational approaches, such as molecular docking and molecular dynamics simulations, offer indispensable alternatives [55]. These techniques provide cost-effective and precise methods to explore the interaction dynamics between radio-iodinated compounds and target enzymes, like AKT1 [56]. The main objective of this study is to leverage these computational strategies to assess the binding affinity and structural integrity of [^125^I]anastrozole and [^125^I]epirubicin in complex with the AKT1 enzyme. By elucidating the binding mechanisms involved, this research aims to underscore the potential of computational methodologies in expediting the development of targeted imaging agents for solid tumors implicating the AKT1 pathway.

## 2. Results

### 2.1. Molecular Docking

**Figure 2:**
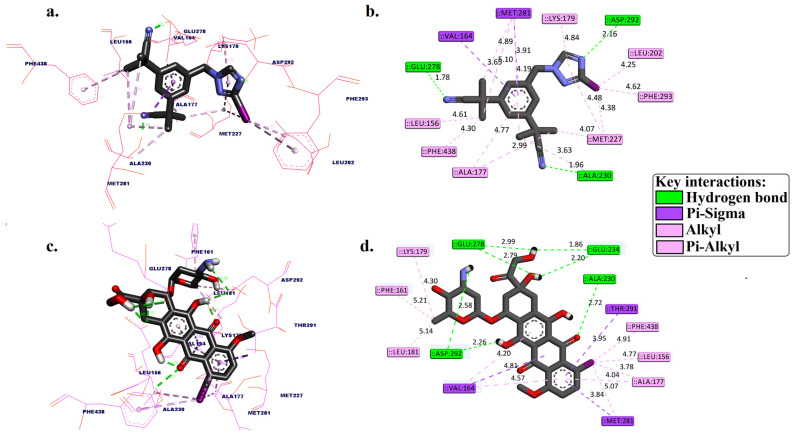
Three-dimensional and 2D binding pose interactions between [^125^I]anastrozole (**a**,**b**) and [^125^I]epirubicin (**c**,**d**) into the active binding site of the human AKT1 enzyme (PDB ID: 3OCB).

In this study, molecular docking simulations were conducted for [^125^I]anastrozole and [^125^I]epirubicin, along with the co-crystallized ligand ((2S)-2-(4-chlorobenzyl)-3-oxo-3-[4-(7H-pyrrolo [2,3-d]pyrimidin-4-yl)piperazin-1-yl]propan-1-amine), targeting the human AKT1 enzyme (PDB ID: 3OCB). The results are summarized in Figure 2 and Table 1.

### 2.2. Molecular Dynamic Simulation

**Figure 3:**
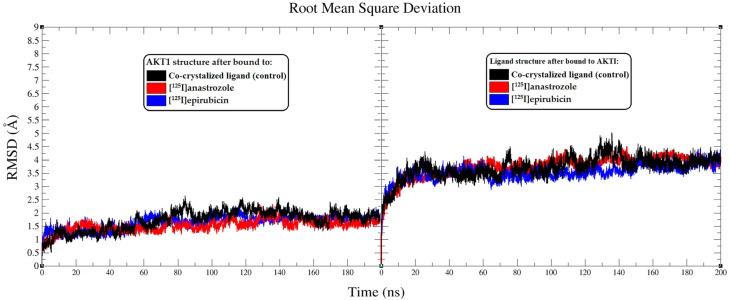
Analysis of root mean square deviation (RMSD) from the molecular dynamics (MD) simulations conducted over a 200 ns period. The left panel presents RMSD plots showing the molecular fluctuations in the AKT1 enzyme backbone when interacting with the co-crystallized ligand (black), [^125^I]anastrozole (red), and [^125^I]epirubicin (blue). The RMSD plots further illustrate the conformational shifts of the co-crystallized ligand (black), [^125^I]anastrozole (red), and [^125^I]epirubicin (blue) as they bind to the AKT1 enzyme.

**Figure 4:**
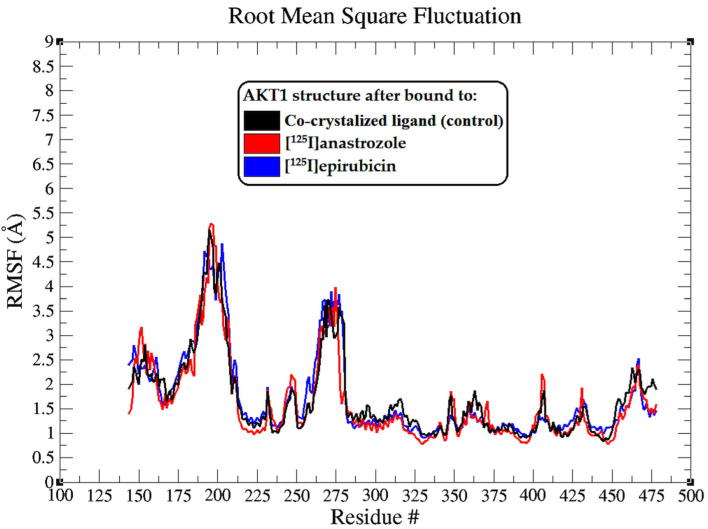
The root mean square fluctuation (RMSF) plots illustrate the fluctuations in the AKT1 backbone atoms over a 200 ns MD simulation for each system. These RMSF values capture the extent of atomic movement for each enzyme residue as its interact with the ligands throughout the simulation.

**Figure 5:**
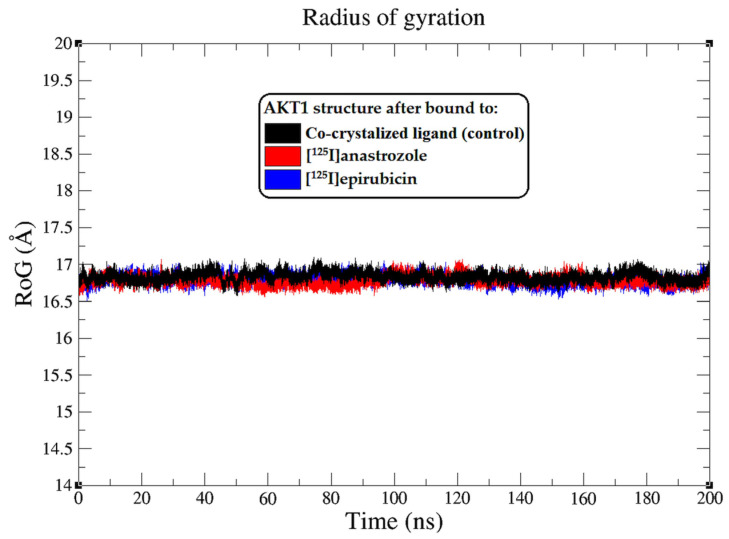
Radius of gyration (RoG) plots showing the AKT1 enzyme backbone atoms throughout the 200 ns MD simulation, illustrating the interactions with the co-crystallized ligand (black), [^125^I]anastrozole (red), and [^125^I]epirubicin (blue).

**Figure 6:**
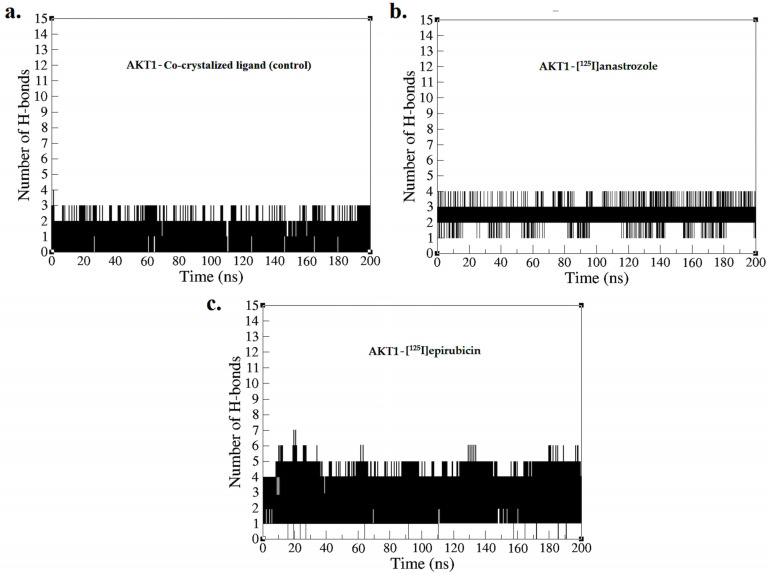
Hydrogen bond profiles obtained from the MD simulation over a time period of 0–200 ns for (**a**) AKT1-co-crystallized ligand, (**b**) AKT1-[^125^I]anastrozole, and (**c**) AKT1-[^125^I]epirubicin.

The interactions between [^125^I]anastrozole and [^125^I]epirubicin with the human AKT1 enzyme were examined through a 200 ns molecular dynamics (MD) simulation using the GROMACS 2016 software suite. This analysis also included the co-crystallized ligand for comparison. Critical metrics, including the root mean square deviation (RMSD), root mean square fluctuation (RMSF), radius of gyration (Rg), and hydrogen bond profiles, were thoroughly analyzed. Furthermore, MM-PBSA calculations were conducted to evaluate the interactions between the ligands and the backbone atoms of the enzyme. The detailed results are illustrated in Figure 3, Figure 4, Figure 5 and Figure 6 and are summarized in Table 2.

## 3. Discussion

### 3.1. Molecular Docking Simulation

Molecular docking is an essential computational technique for predicting the interaction between small molecules and their target proteins, forming stable complexes [57]. This method provides critical insights into binding orientations and affinities, playing a pivotal role in drug discovery by enabling the investigation of potential inhibitory effects on specific target enzymes [58,59,60,61,62]. In our study, the redocking of the co-crystallized served as a validation step for the docking parameters, ensuring that the computational model faithfully replicated the experimentally observed orientation of the ligand within the AKT1 active site [63].

Figure S1 demonstrates the effectiveness of the docking process, with panel (a) displaying the overlay of the co-crystallized ligand and the redocked ligand, highlighting the precision with which the redocking process replicated the original ligand orientation within the binding pocket [58,59,60,64,65]. The root mean square deviation (RMSD) of 0.98 Å serves as a quantitative measure of this comparison, indicating a high degree of conformational similarity between the two poses. Panels (b) and (c) in Figure S1 dissect the interaction profiles of the co-crystallized and redocked ligands, respectively. The diagrams detail key interactions, including hydrogen bonds, pi–sigma interactions, and hydrophobic interactions, which contribute to the stabilization of the ligand within the enzymatic cleft. Notably, the hydrogen bond between the ligand and ALA230 is maintained at a distance of 1.98 Å in the co-crystallized ligand, and a slightly longer distance of 2.67 Å in the redocked ligand, indicating a minor deviation in binding interaction post-redocking. The preservation of critical interactions, such as the hydrophobic interactions with VAL164 and MET281, demonstrates the redocking’s ability to emulate the original binding mode. The interaction distances in the redocked ligand, ranging from 2.16 Å to 2.99 Å, are within acceptable limits compared to the co-crystallized ligand, affirming the reliability of the software used for the docking process [64,65,66,67,68,69,70]. Given the RMSD value of 0.98 Å, the redocking results fall within the generally accepted range for high-quality docking simulations, as established by the literature standards [64,65,66,67,68,69,70]. This degree of precision suggests that the docking software can be confidently applied to similar molecular investigations, such as the study of [^125^I]anastrozole and [^125^I]epirubicin, and the co-crystallized ligand against the AKT1 enzyme.

Figure 2 and Table 1 present a detailed analysis of the interactions involving [^125^I]anastrozole, [^125^I]epirubicin, and the co-crystallized ligand within the active binding site of AKT1. This analysis examines each molecule’s binding affinity and interaction profile, focusing on key interactions, such as hydrogen bonds, hydrophobic interactions, and specifically, the interactions of the ^125^I atom with the enzyme, which is crucial for understanding the mode of binding. [^125^I]anastrozole exhibits a binding affinity with a Δ*G*_bind_ of −10.68 kcal/mol. It forms hydrogen bonds with ALA230, GLU278, and ASP292 at distances of 1.96 Å, 1.78 Å, and 2.16 Å, respectively. Additionally, it engages in pi–sigma interactions with VAL164 and MET281. Extensive hydrophobic interactions are noted with residues such as LEU156, ALA177, LYS179, LEU202, MET227, MET281, PHE293, and PHE438, which are crucial for non-polar binding complementarity. The interaction of ^125^I with specific residues further suggests a competitive and reversible binding mode, likely contributing to the stabilization of the AKT1-ligand complex.

[^125^I]epirubicin shows the most favorable binding affinity with a Δ*G*_bind_ of −11.84 kcal/mol. It forms a network of hydrogen bonds with ALA230, GLU234, GLU278, and ASP292 at distances ranging from 1.86 Å to 2.99 Å. In addition, it engages in pi–sigma interactions with VAL164, MET281, and THR291, alongside extensive hydrophobic interactions involving residues such as LEU156, PHE161, VAL164, ALA177, LYS179, LEU181, and PHE348. The interactions of ^125^I with the enzyme suggest a highly specific binding mode, potentially leading to effective inhibition through competitive and reversible binding mechanisms. The co-crystallized ligand, with a Δ*G*_bind_ of −9.53 kcal/mol, forms hydrogen bonds with ALA230 and GLU278 at distances of 1.98 Å and 2.67 Å, respectively, but does not form pi–sigma interactions. It also engages in hydrophobic interactions with VAL164, ALA177, LYS179, ALA230, and MET281. The absence of iodine in this ligand precludes any direct comparison regarding iodine-specific interactions, but the overall binding profile highlights its role as a control for comparison.

Comparatively, [^125^I]anastrozole and [^125^I]epirubicin establish additional hydrogen bonds and specific ^125^I interactions when compared to the co-crystallized ligand, potentially indicating stronger or additional points of enzyme engagement. This evidence suggests that [^125^I]anastrozole and [^125^I]epirubicin are promising candidates as AKT1 enzyme inhibitors, likely acting through competitive and reversible binding. However, while the binding energies and static interaction profiles are informative, they provide only a snapshot of a dynamic process. To gain a comprehensive understanding of the behavior and stability of these molecules at the molecular level, molecular dynamics simulations are essential. Such simulations will provide temporal insights into the flexibility, conformational changes, and resilience of the binding interactions under physiological conditions, which are critical parameters for the successful development of therapeutic agents.

### 3.2. Molecular Dynamic Simulation

The stability and conformational dynamics of the AKT1 enzyme when complexed with [^125^I]anastrozole, [^125^I]epirubicin, and the co-crystallized ligand were explored over a 200 ns simulation period. To assess structural dynamics, root mean square deviation (RMSD) analyses were performed, focusing on how each ligand influenced the conformational stability of AKT1 [71,72]. The RMSD trajectories for the AKT1 enzyme backbone displayed distinct stability patterns depending on the ligand involved (Figure 3). The complex with the co-crystallized ligand stabilized at an average RMSD value of approximately 2.69 Å after around 87 ns, reflecting consistent enzyme structure throughout the simulation. This stability provides a benchmark for evaluating the investigational ligands. Comparatively, the AKT1 enzyme exhibited greater stability in complexes with [^125^I]anastrozole and [^125^I]epirubicin, with both systems achieving stability after approximately 68 ns and maintaining an RMSD value near 2.2 Å. This suggests that these radio-iodinated ligands contribute to a more stable interaction within the enzyme’s active site compared to the co-crystallized ligand. The RMSD fluctuations for the enzyme in these complexes were within a narrower range, less than 1.3 Å, indicating robust structural alignment and reduced conformational variability.

The RMSD values of the ligands themselves provide further insights into their conformational stability within the AKT1 binding site. Both [^125^I]anastrozole and [^125^I]epirubicin exhibited minimal conformational changes, with RMSD fluctuations averaging less than 1 Å. This consistent stability underscores their potential fit and effectiveness within the active site, suggesting that these radio-iodinated compounds maintain a high degree of structural integrity when bound to AKT1. The stable RMSD values reflect a strong and specific interaction, which is crucial for effective inhibition [73]. These RMSD data underscore the varying stability profiles of the AKT1-ligand complexes. Both [^125^I]anastrozole and [^125^I]epirubicin demonstrated significant conformational stability within the AKT1 binding site, indicating a strong potential to sustain interactions essential for effective enzyme inhibition. The stability observed with the co-crystallized ligand, while slightly less robust, still reinforces its potential as an AKT1 inhibitor, though with more variability in interaction dynamics compared to the radio-iodinated compounds.

The root mean square fluctuation (RMSF) analysis extends our understanding of the dynamic interplay between the AKT1 enzyme and the bound ligands, highlighting the flexibility of particular amino acid residues upon ligand binding [74]. The RMSF profiles, as depicted in Figure 4, are essential for pinpointing regions within the protein that show notable mobility, which could be critical for AKT1’s function and interaction with ligands. Interestingly, the RMSF plots showed that there were no significant shifts in the residues or segments of the enzyme, and all ligands affected the mobility of the residues in a consistent pattern, with differences of less than 2 Å. This suggests that the overall flexibility of the AKT1 enzyme is preserved, regardless of the ligand binding, indicating a stable interaction profile across all tested ligands.

In this comprehensive analysis, the RoG was evaluated as an indicator of the enzyme’s structural compactness upon ligand binding [74]. The Rg plots, illustrated in Figure 5, depict the Rg values of the AKT1 backbone atoms throughout the 0–200 ns MD simulation. The average Rg values offer a quantitative assessment of the overall shape and compactness of the protein–ligand complexes. The AKT1 enzyme, when complexed with the co-crystallized ligand, maintained an average RoG of approximately 16.8 ± 0.1 Å, suggesting a tightly packed protein structure. Similarly, the AKT1 complexes with [^125^I]anastrozole and [^125^I]epirubicin exhibited consistent average RoG values of around 16.8 ± 0.1 Å, indicating that these ligands do not significantly alter the compactness of the enzyme structure. The RMSF and RoG analyses provide additional evidence supporting the high stability and strong interaction profiles of [^125^I]anastrozole and [^125^I]epirubicin within the AKT1 binding site. These findings contribute to the growing body of data suggesting that these radio-iodinated compounds could serve as potent and specific inhibitors of the AKT1 enzyme, with potential applications in targeted breast cancer therapy.

Figure 6 depicts the number of hydrogen bonds formed between the ligands and the AKT1 enzyme during the simulation, providing a quantitative assessment of interaction strength and stability, which is essential for evaluating the efficacy of these compounds as inhibitors [75]. The AKT1 complex with the co-crystallized ligand consistently maintained between two and three hydrogen bonds throughout the duration of the simulation, with two bonds being consistently formed. This result confirms the predicted binding pose and indicates a stable interaction, consistent with the initial docking results presented in Figure 2. [^125^I]anastrozole showed greater consistency in forming hydrogen bonds, fluctuating between three and four bonds, with three bonds being consistently maintained throughout the 200 ns MD simulation. This implies a potentially more effective interaction with AKT1, suggesting that [^125^I]anastrozole may provide enhanced inhibitory effects due to its robust and stable hydrogen bonding. Conversely, [^125^I]epirubicin exhibited the highest number of hydrogen bonds, fluctuating between four and five, with four bonds consistently maintained. The higher number of consistent hydrogen bonds suggests an even stronger and more stable interaction with the AKT1 enzyme, potentially leading to more effective inhibition due to improved positional integrity within the enzyme’s active site. These hydrogen bond profiles from the MD simulations underscore the varying interaction strengths and stabilities across the different ligand-AKT1 complexes. [^125^I]epirubicin, with its greater number of consistent hydrogen bonds, emerges as a particularly promising candidate for further development. The variations observed with the co-crystallized ligand highlight the critical role of optimizing hydrogen bond interactions to enhance ligand efficacy. [^125^I]anastrozole, with its ability to maintain a stable number of hydrogen bonds, also demonstrates potential as an effective AKT1 inhibitor. These results suggest that radio-iodinated compounds, especially [^125^I]epirubicin, could be effective targeted radiopharmaceuticals for breast cancer therapy.

To further support the previous analyses, Molecular Mechanics Poisson–Boltzmann Surface Area (MM-PBSA) calculations were conducted to obtain deeper insights into the binding free energies of the AKT1 complexes with the radio-iodinated compounds [76]. These calculations are crucial for validating the significance of the observed hydrogen bond interactions and for understanding the energetic contributions within the AKT1 enzyme’s active binding site [76]. Table 2 summarizes the MM-PBSA binding energies for the co-crystallized ligand (control), [^125^I]anastrozole, and [^125^I]epirubicin, providing a quantitative perspective on the various energetic factors contributing to the overall binding affinity at the AKT1 active site.

The binding free energy (Δ*G*_bind_) for the AKT1-co-crystallized ligand complex is −16.38 ± 0.14 kcal/mol, which serves as a reference point for comparing the other ligands. The electrostatic contributions for the co-crystallized ligand are substantial at −11.28 ± 0.11 kcal/mol, indicating that electrostatic forces play a significant role in the binding affinity. These are balanced by van der Waals interactions at −13.52 ± 0.12 kcal/mol and an unfavorable polar solvation energy of 19.11 ± 0.12 kcal/mol, offset by a favorable non-polar solvation energy of −10.69 ± 0.13 kcal/mol. The non-polar solvation energy is crucial for stabilizing the ligand within the enzyme’s hydrophobic pockets. [^125^I]anastrozole has a more favorable binding free energy of −20.03 ± 0.15 kcal/mol, indicating a stronger binding affinity than the co-crystallized ligand. This stronger affinity is due to higher electrostatic interactions at −12.86 ± 0.13 kcal/mol and more substantial van der Waals forces at −14.69 ± 0.11 kcal/mol. The elevated electrostatic interaction scores indicate an improved alignment within the active site, thereby enhancing the ligand’s interaction with crucial residues. While the polar solvation penalty is slightly higher at 19.84 ± 0.13 kcal/mol, the favorable non-polar solvation energy of −12.32 ± 0.12 kcal/mol suggests effective accommodation within the enzyme’s non-polar regions. [^125^I]epirubicin shows the most favorable Δ*G*_bind_ of −23.57 ± 0.14 kcal/mol, with significant electrostatic interactions at −14.73 ± 0.12 kcal/mol and notable van der Waals forces at −15.84 ± 0.14 kcal/mol. These contributions indicate a well-balanced interaction dynamic within the active site, resulting in a robust binding affinity. Despite the slightly higher polar solvation energy at 19.86 ± 0.12 kcal/mol, the favorable non-polar solvation energy of −12.86 ± 0.13 kcal/mol supports the ligand’s stable binding. The MM-PBSA binding energy analysis indicates that all ligands possess potential as AKT1 inhibitors, with [^125^I]epirubicin exhibiting the highest binding affinity, closely followed by [^125^I]anastrozole. The favorable binding free energies and substantial electrostatic contributions suggest that these investigational ligands hold promise as effective AKT1 inhibitors.

Overall, the computational analysis demonstrated that [^125^I]epirubicin exhibited the most favorable binding affinity to AKT1, followed closely by [^125^I]anastrozole, surpassing the affinity of the co-crystallized ligand. To contextualize these findings, it is imperative to compare them with the binding affinities and interaction profiles of other known AKT1 inhibitors documented in the literature. Prominent AKT1 inhibitors, such as capivasertib (AZD5363) [77,78] and afuresertib (GSK2110183) [79,80,81], have been extensively studied and have shown significant inhibitory effects on AKT1. For instance, capivasertib has been reported to exhibit binding affinities in the range of −9.0 to −10.0 kcal/mol [82,83], while afuresertib has shown affinities ranging from −8.5 to −9.5 kcal/mol [84,85]. When compared to these established inhibitors, the binding affinity of [^125^I]epirubicin at −11.84 kcal/mol indicates a potentially stronger interaction with the AKT1 kinase domain, suggesting an enhanced therapeutic potential. The interaction profiles of capivasertib and afuresertib commonly involve critical residues within the AKT1 active site, such as THR308, SER473, and ASP292, which are integral to the enzyme’s catalytic function. The findings indicate that [^125^I]epirubicin and [^125^I]anastrozole engage with similar residues, particularly ASP292, thereby implying that these radio-iodinated compounds could effectively inhibit AKT1 by interfering with its phosphorylation and subsequent activation. Notably, the MM-PBSA calculations revealed that [^125^I]epirubicin forms more stable hydrogen bonds and van der Waals interactions compared to traditional AKT1 inhibitors, which may contribute to its superior binding stability. 

However, it is important to recognize that the findings presented in this study are based on computational predictions, which, while informative, are inherently limited by the approximations and assumptions underlying molecular docking and dynamics simulations [58,59,60,64,65]. These methods do not fully capture the complexity of biological systems, including the influence of cellular environments, off-target effects, and metabolic stability. Thus, it is essential to validate these computational predictions with in vitro and in vivo studies to confirm the therapeutic efficacy and safety profiles of these compounds. Experimental studies will be crucial to verifying the investigational ligands’ inhibitory action on AKT1 and determining their suitability for clinical development. Such studies will provide a more comprehensive understanding of the pharmacodynamics, pharmacokinetics, and potential toxicity of [^125^I]anastrozole and [^125^I]epirubicin, which are critical for assessing their viability as therapeutic agents.

## 4. Methodology

### 4.1. Molecular Docking Simulation

In this research, the focus was on the AKT1 enzyme, analyzed for its interaction with the radio-iodinated compounds anastrozole and epirubicin. The selection of AKT1 as the target for this study was based on its critical role in the PI3K/AKT/mTOR signaling pathway, which is frequently dysregulated in breast cancer and contributes to tumor growth and survival [39,40]. The crystallographic details of the AKT1 enzyme in complex with a pyrrolopyrimidine inhibitor ((2S)-2-(4-chlorobenzyl)-3-oxo-3-[4-(7H-pyrrolo [2,3-d]pyrimidin-4-yl)piperazin-1-yl]propan-1-amine) were obtained from the Protein Data Bank (PDB) [86], with the accession number 3OCB [87], (https://www.rcsb.org/structure/3OCB) accessed on 29 April 2024.

In preparation for molecular docking, the enzyme target underwent preprocessing to ensure its suitability for computational analysis. This involved the removal of non-essential water molecules and heteroatoms using Biovia Discovery Studio Visualizer [88], yielding a refined enzyme structure in a PDB format. Missing residues within the enzyme structure were reconstructed using the YASARA web server [71,89,90]. The protonation states of titratable amino acids were calculated at a physiological pH of 7.4 via the H^++^ web server [91]. Subsequently, polar hydrogen atoms were added, and Kollman charges were assigned, facilitating the conversion of the structure to a PDBQT format for subsequent docking simulations using AutoDock Tools version 1.5.6 [92].

The preparation and optimization of ligands for molecular docking simulations were conducted with the objective of maximizing the accuracy and reliability of the resulting computational predictions [93]. Radio-iodinated molecules ([^125^I]anastrozole and [^125^I]epirubicin) were drawn using the ChemDraw JS (v7.1) web server [71], inspired by structures outlined in previous studies [47], and saved in a structural data file (SDF) format. The ligands were subjected to energy minimization utilizing the universal force field and a conjugate gradient optimization algorithm [94]. This procedure, executed over a thousand iterations, was performed using Open Babel software (https://openbabel.org/) [95], resulting in final structures saved in the PDB format. Subsequently, Gasteiger charges were assigned using AutoDock Tools version 1.5.6, preparing the structures for docking simulations in the PDBQT format.

To elucidate the interactions between the ligands and the active site of AKT1, molecular docking was performed using AutoDock 4.2 [92]. A Lamarckian genetic algorithm was employed to optimize ligand configurations within the binding site, allowing flexibility in the ligand while keeping the macromolecule fixed [96]. The grid-box dimensions were set to 40 × 40 × 40 Å for the X, Y, and Z axes, respectively, with central grid point coordinates at 12.916, 0.179, and 17.863. The docking process was conducted over 100 trials with a maximum of 25,000,000 evaluations per run, adhering to default parameters to ensure consistency in the analyses.

### 4.2. Molecular Dynamic Simulation

This study primarily investigated the interaction and stability of [^125^I]anastrozole and [^125^I]epirubicin within the active sites of the AKT1 enzyme using molecular dynamics (MD) simulations over a span of 200 ns. The crystallographic structure utilized for these simulations was the human AKT1 kinase domain complexed with a pyrrolopyrimidine inhibitor ((2S)-2-(4-chlorobenzyl)-3-oxo-3-[4-(7H-pyrrolo [2,3-d]pyrimidin-4-yl)piperazin-1-yl]propan-1-amine), obtained from the Protein Data Bank (PDB ID: 3OCB). The simulations were conducted using the GROMACS 2016.3 software package with the Gromos96 54a7 force field to perform an in-depth analysis of the ligand–protein interactions [97]. Over the 200 ns simulation period, valuable data on binding dynamics and molecular stability within the enzyme’s active sites were gathered.

To initiate the simulations, topology files for both the ligands and the protein were prepared. Ligand topology was generated using the GROMACS ‘pdb2gmx’ function, while protein topology was created via the PRODRG server, accessed on 5 May 2024 [http://davapc1.bioch.dundee.ac.uk/cgi-bin/prodrg]. The systems were subsequently solvated in a TIP3P water model and neutralized with counterions to establish a balanced environment conducive to interaction studies. Energy minimization was then performed using the steepest descent method for 50,000 steps, with each step measured at 0.01 energy units, to ensure optimal system stability [71,72].

The system equilibration process was executed in two phases: initially using the NVT ensemble for 100 ps at a temperature of 310 K with the v-rescale thermostat, followed by the NPT ensemble for another 100 ps at 1.0 bar using the Berendsen pressure coupling method [98,99]. These steps were crucial for ensuring that the systems reached a stable state prior to the molecular dynamics (MD) simulations. The MD simulations were subsequently run for 200 ns under constant conditions of 310 K and 1 bar. A 1.0 nm cut-off was applied for short-range, non-bonded interactions, while long-range, electrostatic interactions were handled using the Particle Mesh Ewald method [100]. To preserve structural integrity, the LINCS algorithm was used to constrain bonds involving hydrogen atoms [101]. The simulations employed a timestep of 2 fs with coordinate data saved every 5000 steps (10 ps), a strategy that ensured accurate and reliable simulation results [71,72].

To assess the fidelity and stability of the ligand–enzyme interactions, a comprehensive analysis of the MD trajectory data was conducted, utilizing metrics such as the root mean square deviation (RMSD), root mean square fluctuation (RMSF), radius of gyration (Rg), and hydrogen bond profiles. This methodological approach reflects a rigorous commitment to obtaining precise and meaningful insights into the molecular dynamics of the systems under investigation.

### 4.3. MM-PBSA Calculation

In this study, the Molecular Mechanics Poisson–Boltzmann Surface Area (MM-PBSA) method was applied to estimate binding free energies based on MD trajectory snapshots [76]. This method, known for its effectiveness in accurately assessing binding energies, enabled a comprehensive analysis of the energetic interactions between the radio-iodinated compounds ([^125^I]anastrozole and [^125^I]epirubicin) and the co-crystallized ligand within the AKT1 enzyme’s active site. MM-PBSA calculations were performed during the production phase of the MD simulations, with snapshots taken at 100 ps intervals over the final 20 ns (from 180 to 200 ns). These calculations were conducted using the g_mmpbsa tool within the GROMACS software package [102,103], allowing for a detailed evaluation of interaction strengths.

This method offered valuable insights into the binding efficiencies of [^125^I]anastrozole and [^125^I]epirubicin relative to the co-crystallized ligand, elucidating the molecular features that could influence their therapeutic potential. The equations used for calculating the binding free energy of the ligand–enzyme complex in solvent, which form the foundation of the MM-PBSA method, have been extensively documented in the existing literature [71,72].

## 5. Conclusions

This study represents the first comprehensive in silico exploration of the binding potential of radioiodinated anastrozole ([^125^I]anastrozole) and epirubicin ([^125^I]epirubicin) against the AKT1 enzyme, a critical target in breast cancer therapy. Utilizing a range of computational techniques, including molecular docking, molecular dynamics simulations (200 ns), and MM-PBSA calculations, the therapeutic potential of these compounds was thoroughly evaluated. Molecular docking results indicate that [^125^I]epirubicin displays the highest binding affinity to AKT1 with a Δ*G*_bind_ of −11.84 kcal/mol, followed by [^125^I]anastrozole at -10.68 kcal/mol, and the co-crystallized ligand at −9.53 kcal/mol. These findings suggest that [^125^I]epirubicin may provide superior inhibitory effects against AKT1 due to its stronger molecular interactions within the active site. Molecular dynamics simulations further corroborated these results, showing that both [^125^I]anastrozole and [^125^I]epirubicin formed stable complexes with AKT1, as reflected by RMSD values consistently around 2.2 Å, while the co-crystallized ligand stabilized at approximately 2.69 Å. RMSF and radius of gyration analyses revealed minimal disruption to the enzyme’s overall structure and dynamics, which is crucial for preserving its biological function. Specifically, the consistent RMSF patterns and average Rg values (~16.8 ± 0.1 Å) suggest that these ligands do not significantly alter the enzyme’s compactness or flexibility. Additionally, MM-PBSA calculations supported the findings from docking and dynamics simulations, confirming that [^125^I]epirubicin exhibits the most favorable binding energy (−23.57 ± 0.14 kcal/mol), driven by significant electrostatic and van der Waals interactions. The binding free energies of [^125^I]anastrozole and the co-crystallized ligand also indicated their potential as AKT1 inhibitors, although to a slightly lesser degree compared to [^125^I]epirubicin. This study highlights the promising inhibitory potential of [^125^I]anastrozole and [^125^I]epirubicin, particularly [^125^I]epirubicin, against AKT1, suggesting that these compounds could serve as valuable leads in the development of new therapeutic agents for breast cancer. Future research should focus on empirical validation through in vitro and in vivo testing to confirm these computational predictions and fully assess the therapeutic efficacy and safety profiles of these compounds. Planned studies include cell-based assays to evaluate cytotoxicity and enzyme inhibition, as well as animal models to assess pharmacokinetics, biodistribution, and therapeutic outcomes. The broader impact of these findings lies in their contribution to the field of radiopharmaceuticals and personalized cancer therapy. By demonstrating the potential of [^125^I]anastrozole and [^125^I]epirubicin as AKT1-targeted radiopharmaceuticals, this study advances the development of novel diagnostic and therapeutic agents that can be tailored to the molecular profile of individual tumors, thereby enhancing treatment specificity and efficacy. Such advancements could pave the way for more effective and personalized treatment strategies in oncology, particularly in managing breast cancer.

## Figures and Tables

**Table 1:** Molecular docking scores, expressed as free binding energy (kcal/mol), for [^125^I]anastrozole (a & b), [^125^I]epirubicin, and the co-crystallized ligand, targeting the human AKT1 enzyme. The table also provides a detailed analysis of the 2D molecular interactions between these compounds and the key residues within the AKT1 active site (PDB ID: 3OCB).

Compounds	Free Binding Energy (kcal/mol)	Molecular Interactions Analysis within the AKT1 Active Binding Site			
		H-bond	Distance (Å)	Pi-Sigma	Hydrophobic Interaction
[^125^I]anastrozole	−10.68	ALA230, GLU278, and ASP292	1.96, 1.78, and 2.16	VAL164 and MET281	LEU156, ALA177, LYS179, LEU202, MET227, MET281, PHE293, and PHE438
[^125^I]epirubicin	−11.84	ALA230, GLU234, GLU234, GLU278, GLU278, ASP292, and ASP292	2.72, 1.86, 2.20, 2.79, 2.99, 2.26, and 2.58	VAL164, MET281, and THR291	LEU156, PHE161, VAL164, ALA177, LYS179, LEU181, PHE281, and PHE348
* Co-crystalized ligand (original pose)	−9.53	ALA230 and GLU278	1.98 and 2.67	-	VAL164, ALA177, LYS179, ALA230, and MET281

* Co-crystalized ligand: (2S)-2-(4-chlorobenzyl)-3-oxo-3-[4-(7H-pyrrolo [2,3-d]pyrimidin-4-yl)piperazin-1-yl]propan-1-amine.

**Table 2:** MM-PBSA binding energies (Δ*G*_bind_) of the co-crystallized ligand (control), [^125^I]anastrozole, and [^125^I]epirubicin at the active binding site of the AKT1 enzyme (PDB ID: 3OCB). Energy values are expressed in kcal/mol.

System	Δ*G*_bind_ (kcal/mol)	Electrostatic (kcal/mol)	Van der Waal (kcal/mol)	Polar Salvation (kcal/mol)	Non-Polar Salvation (kcal/mol)
AKT1-Co-crystallized ligand	−16.38 ± 0.14	−11.28 ± 0.11	−13.52 ± 0.12	19.11 ± 0.12	−10.69 ± 0.13
AKT1-[^125^I]anastrozole	−20.03 ± 0.15	−12.86 ± 0.13	−14.69 ± 0.11	19.84 ± 0.13	−12.32 ± 0.12
AKT1-[^125^I]epirubicin	−23.57 ± 0.14	−14.73 ± 0.12	−15.84 ± 0.14	19.86 ± 0.12	−12.86 ± 0.13

## Data Availability

Data are contained in manuscript and Supplementary Materials.

## Supplementary Materials

The following supporting information can be downloaded at: https://www.mdpi.com/article/10.3390/molecules29174203/s1, Figure S1. Radio-iodinated anastrozole (a) and epirubicin (b). Figure S2. Superimposition (a) and 2D interactions analysis of the co-crystallized ligand (green C, red O, and blue N) (b) and redocked ligand (orange C, red O, and blue N) (c). The crystal structure of human Akt1 kinase domain in complex with pyrrolopyrimidine inhibitor (3OCB.pdb) (RMSD is ~0.98 Å).
