# Supplementary material for: Exploring Radioiodinated Anastrozole and Epirubicin as AKT1-Targeted Radiopharmaceuticals in Breast Cancer: In Silico Analysis and Potential Therapeutic Effect with Functional Nuclear Imagining Implications
Source: Molecules. 2024 Sep 4;29(17):4203. doi: 10.3390/molecules29174203 (PMC11397058; doi:10.3390/molecules29174203)
Supplement: Supplementary file 1 [file molecules-29-04203-s001.zip › molecules-3166995-supplementary.pdf]

Supplementary Materials

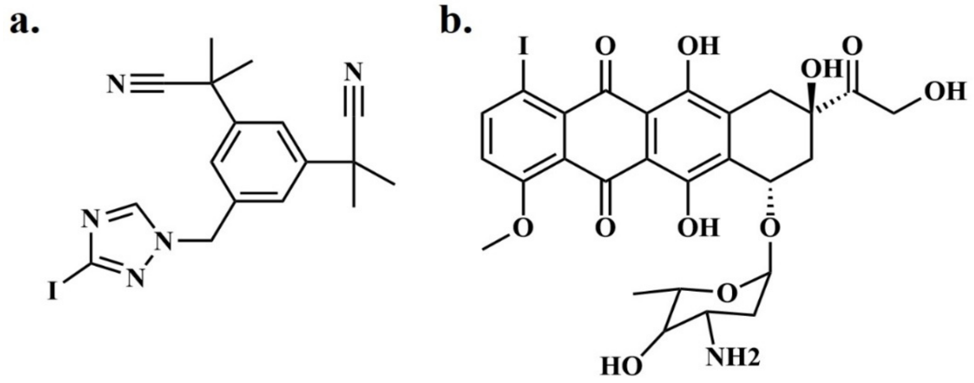

Figure S1. Radioiodinated anastrozole (a) and epirubicin (b).

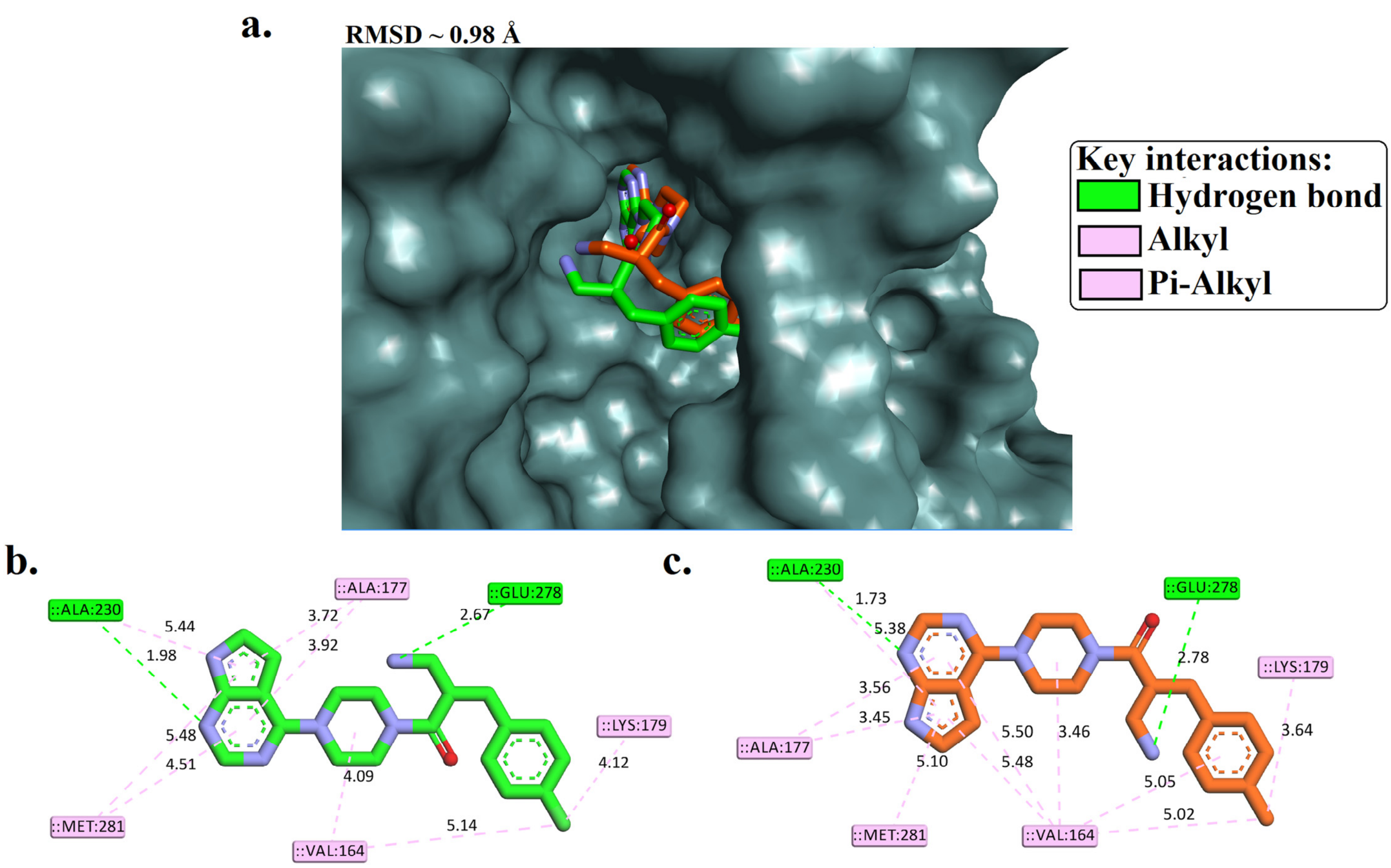

Figure S2. Superimposition (a) and 2D interactions analysis of the co-crystallized ligand (green C, red O, and blue N) (b) and re-docked ligand (orange C, red O, and blue N) (c). The crystal structure of human Akt1 kinase domain in complex with pyrrolopyrimidine inhibitor (3OCB.pdb) (RMSD is ~0.98 Å).
